# Synthesis and crystal structure of tetra­kis­(ethyl­ammonium) hepta­iodide(3−) iodide

**DOI:** 10.1107/S2056989026010844

**Published:** 2026-10-30

**Authors:** Dmytro A. Haleliuk, Mihaela Silion, Dina D. Naumova, Irina A. Golenya, Il’ya A. Gural’skiy

**Affiliations:** a Department of Chemistry, Taras Shevchenko National University of Kyiv, Volodymyrska str. 64/13, 01601 Kyiv, Ukraine; b Department of Physics of Polymers and Polymeric Materials, "Petru Poni", Institute of Macromolecular Chemistry, Iasi, Romania; Vienna University of Technology, Austria

**Keywords:** crystal structure, polyiodide, hepta­iodide(3–), halogen bonding, ethyl­ammonium, N—H⋯I hydrogen bonds

## Abstract

The crystal structure of the title salt, 4C_2_H_5_NH_3_^+^·I_7_^3−^·I^−^, comprises disordered ethyl­ammonium cations accompanied by an isolated and disordered I^−^ anions and [I_7_]^3–^ polyiodide anions. The positional disorder of the ethyl­ammonium cations and isolated I^−^ anions is caused by the crystallographically imposed mirror symmetry. The entire [I_7_]^3–^ polyanion exhibits point group symmetry 2/*m* and is composed of two neutral I_2_ mol­ecules acting as connectors between three I^−^ anions. The hepta­iodide anion has a unique linear conformation and forms a slipped-stack layered arrangement along the *c* axis. The organic cations are accommodated between the anionic units and are linked to them *via* N—H⋯I hydrogen bonds, establishing a supra­molecular framework. As a novel polyiodide compound, the reported structure expands the known structural diversity and topologies of complex iodine-based systems.

## Abstract 2

The crystal structure of 4C_2_H_5_NH_3_^+^·I_7_^3–^·I^−^ comprises disordered ethyl­ammonium cations, an isolated disordered I^−^ anion and a linear [I_7_]^3–^ polyiodide anion. The crystal packing is consolidated by N—H⋯I inter­actions between cations and the anions.

## 1. Chemical context

Halogens are characterized by remarkable chemical versatility, particularly their ability to form a wide variety of polyhalide anions. Within this class, iodine-based anions have maintained a prominent position in chemical research. A major structural milestone was achieved in 1933 with the first complete crystallographic determination of a polyiodide, ammonium triiodide, (NH_4_)I_3_ (Mooney, 1935 ▸). Up until now, a large number of polyiodide anions have been structurally characterized, establishing an impressive range of anions from I_2_^−^ to I_29_^3–^. Their rich chemistry has been thoroughly summarized in several comprehensive reviews (Svensson & Kloo, 2003 ▸; Sonnenberg *et al.*, 2020 ▸). This fascinating structural diversity manifests in structural units ranging from discrete anions to complex one-, two-, and three-periodic extended networks.

The great structural variety stems from the ability of iodine atoms to form chains via halogen bonds, a process further shaped by the surrounding counter-ions. These structural features have been rationalized and utilized in template synthesis, as well as in mol­ecular and crystal engineering, where different cations are used to achieve specific structural topologies. Furthermore, polyiodides attract continuing inter­est due to their diversified properties, which allow for useful applications in a variety of areas, including superconductors, solar cells, and optical devices (Küpper *et al.*, 2011 ▸).

In the present article, we report the synthesis and crystal structure analysis of a new polyiodide compound, 4(C_2_H_5_NH_3_)^+^·I_7_^3–^·I^−^.
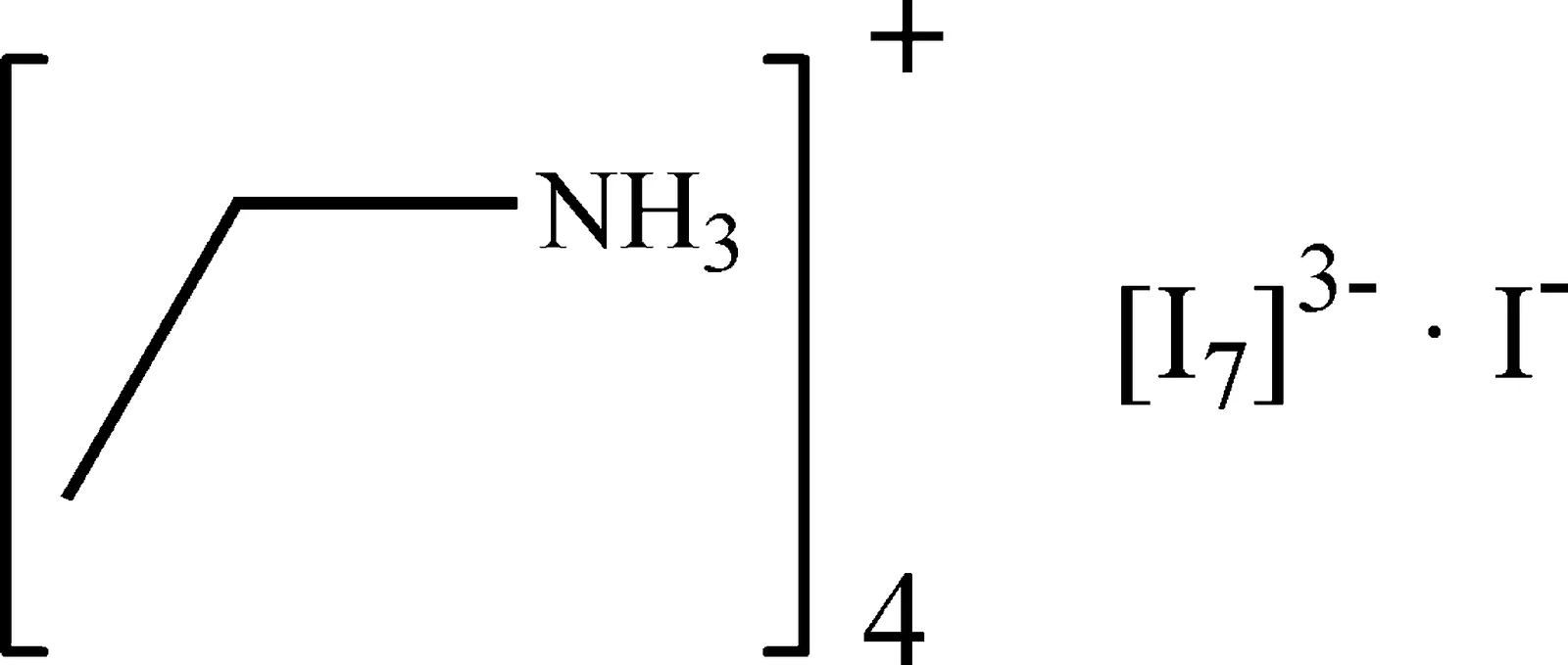


## 2. Structural commentary

The title compound crystallizes in the monoclinic space group *C*2*/m*, with two ethyl­ammonium cations and five I atoms in the asymmetric unit. In the extended structure, isolated I^−^ and polyiodide [I_7_]^3–^ anions are present. The positional disorder (Fig. 1 ▸) for the two cations is caused by the imposed mirror symmetry and that of the isolated anion (I5) by its special position (2/*m*; multiplicity 2, Wyckoff letter *a*).

Selected I—I bond lengths and angles of the [I_7_]^3–^ polyanion are summarized in Table 1 ▸. The polyiodide can be described as two neutral I_2_ mol­ecules (I2, I3) acting as connectors between three I^−^ anions (I1, I4 and its symmetry-related counterpart) in a linear manner. The special position (2 *c*) of the central I1 atom results in point group symmetry 2/*m* for the entire polyanion. The I2—I3 distance is slightly longer than that in an elemental iodine mol­ecule (2.72 Å; Svensson & Kloo, 2003 ▸). This is due to the n→σ* character of the halogen bond, which typically elongates the covalent bond of an I_2_ mol­ecule. The distances between these I_2_ mol­ecules and the three iodide anions (I1—I2 and I3—I4) are significantly longer. For comparison, similar I—I distances [2.8127 (5), 3.4270 (6) and 3.3875 (7) Å] are observed in [HMTAHD]^3+^[I_7_]^3–^ (HMTAHD = 2,2,9,9,16,16-hexa­methyl-2,9,16-triazoniahepta­decane; Lin *et al.*, 2012 ▸). As seen in Table 1 ▸, the angles between the I atoms in the [I_7_]^3–^ anion are all close to 180°. This polyiodide species adopts a unique linear conformation that has not been previously reported.

The negative charge of I^−^ and [I_7_]^3–^ anions is balanced by four ethyl­ammonium cations. The C—C bond lengths are 1.522 (9) Å and 1.525 (11) Å and the C—N bond lengths are 1.478 (6) and 1.423 (10) Å, which are within the expected ranges for ethyl­ammonium cations (Allen *et al.*, 1987 ▸).

## 3. Supra­molecular features

The shortest I⋯I distances between the isolated I^−^ and [I_7_]^3–^ anions are 4.5891 (5) Å [I5*A*⋯I2^i^; symmetry code: (i) −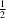 + *x*, −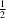 + *y*, *z*], 4.6691 (3) Å (I5*A*⋯I1^i^) and 5.3798 (4) Å (I5*A*⋯I4), which significantly exceed the sum of the van der Waals radii of 4.3 Å for two iodine atoms (Svensson & Kloo, 2003 ▸), indicating no significant covalent or non-covalent inter­actions between them. The layers of polyiodide anions exhibit a slipped-stack arrangement along the *c* axis (Fig. 2 ▸). The organic cations are located between the anionic chains and are linked to them *via* N—H⋯I hydrogen bonds (Fig. 3 ▸, Table 2 ▸).

## 4. Database survey

A search in the Cambridge Crystal Database (CSD, version 5.43, last update May 2026; Groom *et al.*, 2016 ▸) for hepta­iodide moieties, including both [I_7_]^−^ and [I_7_]^3–^ anions, revealed 33 entries. Among these, only two structures contain an additional, isolated I^−^ anion co-crystallized alongside the hepta­iodide moiety (JAPRUO, Antoniadis *et al.*, 2005 ▸; ONOCOM, Agarwal, 2021 ▸).

To highlight the structural novelty of the title compound, it is compared with the closest discrete hepta­iodide analogues: hexa­kis­(2-hy­droxy­pyridine) bis­(2-hy­droxy­pyridinium) hemikis(iodide) sesquikis(hepta­iodide) diiodine (JAPRUO; Antoniadis *et al.*, 2005 ▸) and 1,3-dimethyl-1*H*-imidazol-3-ium hepta­iodide (YUPXOY; Fei *et al.*, 2015 ▸). It is also worth noting the already mentioned structure of [HMTAHD]^3+^[I_7_]^3–^ (PEKKEX; Lin *et al.*, 2012 ▸). The three compounds exhibit significant structural differences compared to the linear [I_7_]^3–^ conformation of the title compound. In both JAPRUO and YUPXOY, the [I_7_]^−^ anions adopt bent (zigzag or Z-type) shapes described as I_2_·I_3_^−^·I_2_ adducts featuring short I—I bonds typical for I_2_ subunits. The only other [I_7_]^3–^ species, PEKKEX, shares the same I^−^·I_2_·I^−^·I_2_·I^−^ sequence. However, this polyiodide anion shows a longer I_2_ covalent bond of 2.8127 (5) Å and adopts a V-shaped arrangement with an I⋯I⋯I angle of 136.22 (9)°.

## 5. Synthesis and crystallization

Ethyl­amine (0.5 mmol) was mixed with iodine (1 mmol) in 3 ml of 55%_wt_ HI. The mixture was stirred for 4 h and then left to stand for one month. Black crystals formed during this period. The crystals were separated and stored under Paratone oil until the diffraction experiment.

## 6. Refinement

Crystal data, data collection and structure refinement details are summarized in Table 3 ▸. The I5 atom representing the isolated iodide anion is disordered over a special position with site symmetry 2/*m* and was refined with occupancies of 0.5 for I5*A* and 0.25 for I5*B* to ensure a total charge of −1. The ethyl­ammonium group N1—C1—C2 lies in a mirror plane; the hydrogen atoms attached to N1 and C2 are disordered around this mirror plane and were refined with an occupancy of 0.5 each. In the ethyl­ammonium group N2—C3—C4, the C4 atom lies on a mirror plane; atoms N2 and C3, as well as all H atoms bonded to them, are disordered about this mirror plane and thus were refined with an occupancy of 0.5. The H atoms were positioned geometrically and were allowed to ride on their parent atom with *U*_iso_(H) = 1.5*U*_eq_(C) for methyl H atoms, *U*_iso_(H) = 1.2*U*_eq_(C) for methyl­ene H atoms, and *U*_iso_(H) = 1.2*U*_eq_(N) for ammonium H atoms.

## Supplementary Material

Crystal structure: contains datablock(s) I. DOI: 10.1107/S2056989026010844/wm5816sup1.cif

Structure factors: contains datablock(s) I. DOI: 10.1107/S2056989026010844/wm5816Isup2.hkl

CCDC reference: 2596264

Additional supporting information:  crystallographic information; 3D view; checkCIF report

## Figures and Tables

**Figure 1:**
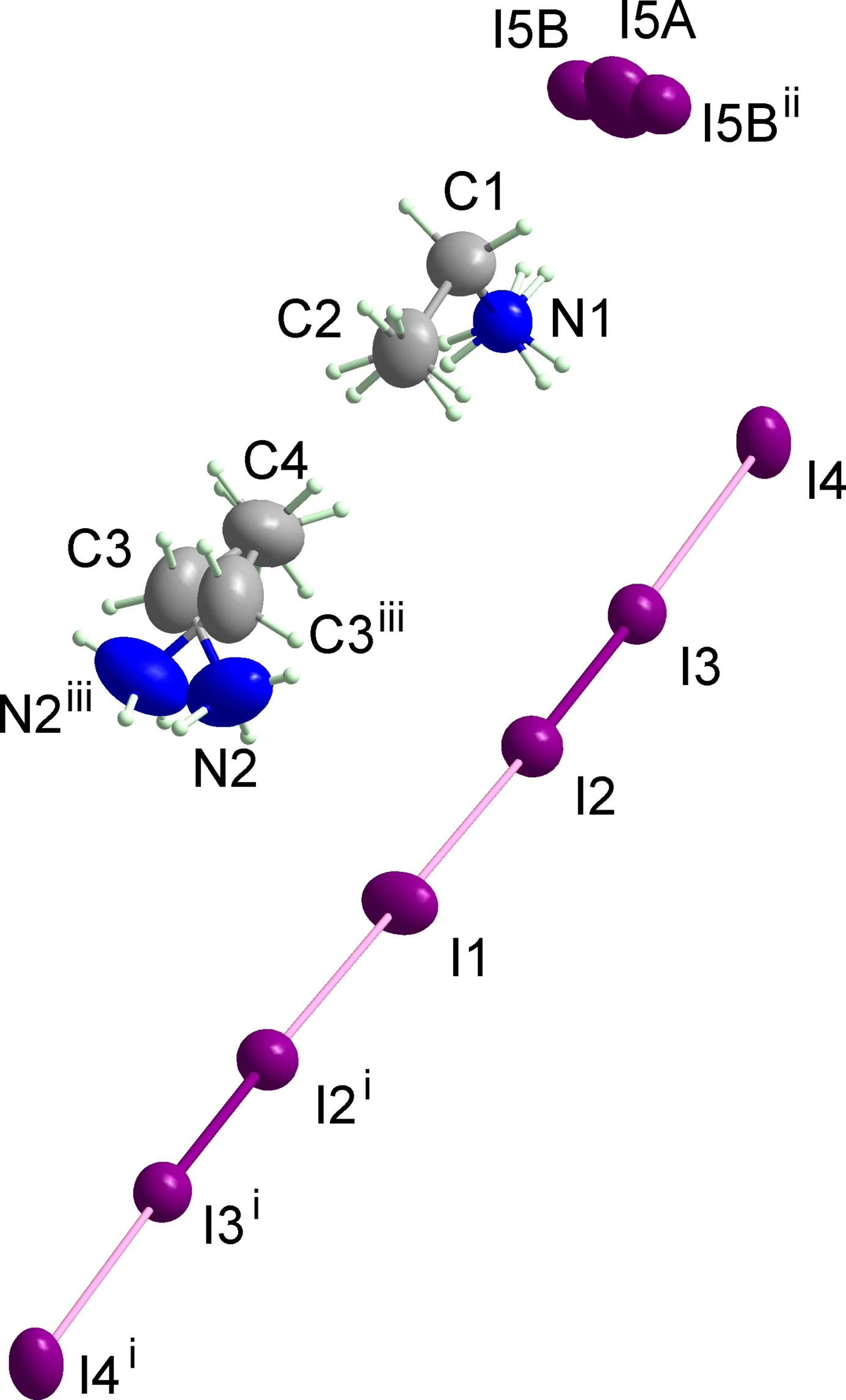
The principal structural units in the crystal structure of the title compound. Displacement ellipsoids are drawn at the 50% probability level; short I—I bonds are drawn as dark solid lines, longer I—I bonds as bright solid lines. [Symmetry codes: (i) 2 − *x*, *y*, 1 − *z*; (ii) 1 − *x*, 1 − *y*, 2 − *z*; (iii) *x*, 1 − *y*, *z*.]

**Figure 2:**
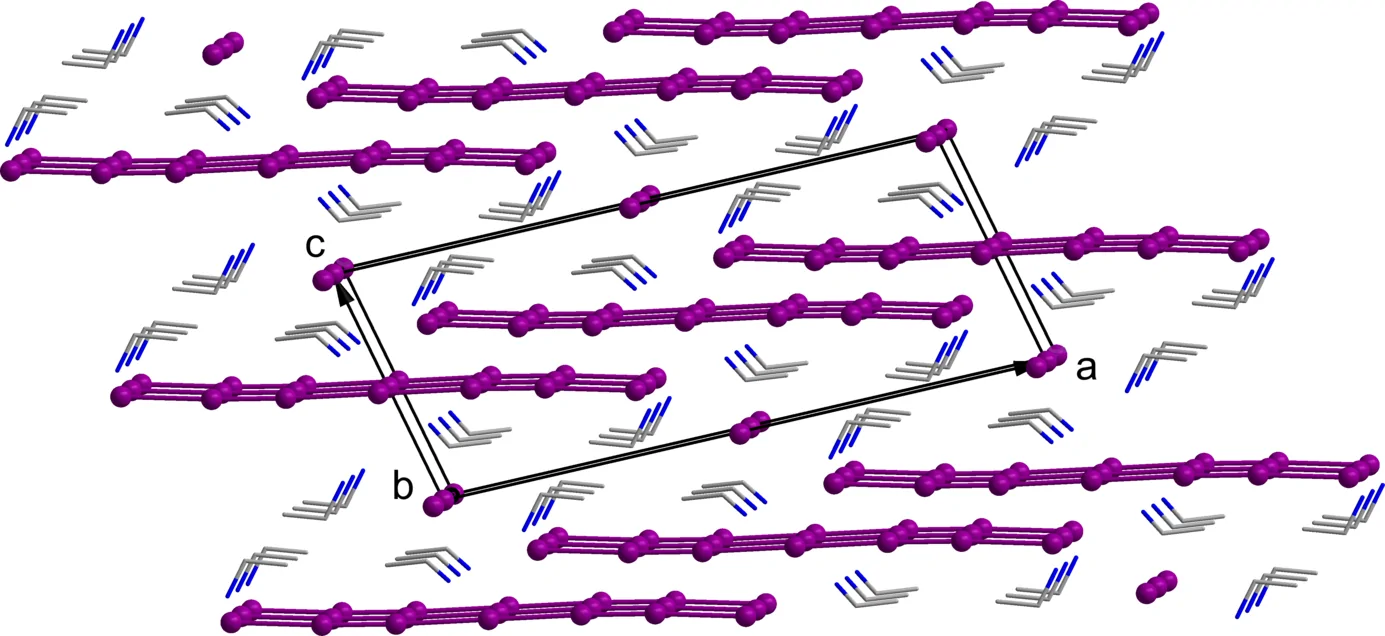
The crystal structure of the title compound in a view approximately along the *b* axis.

**Figure 3:**
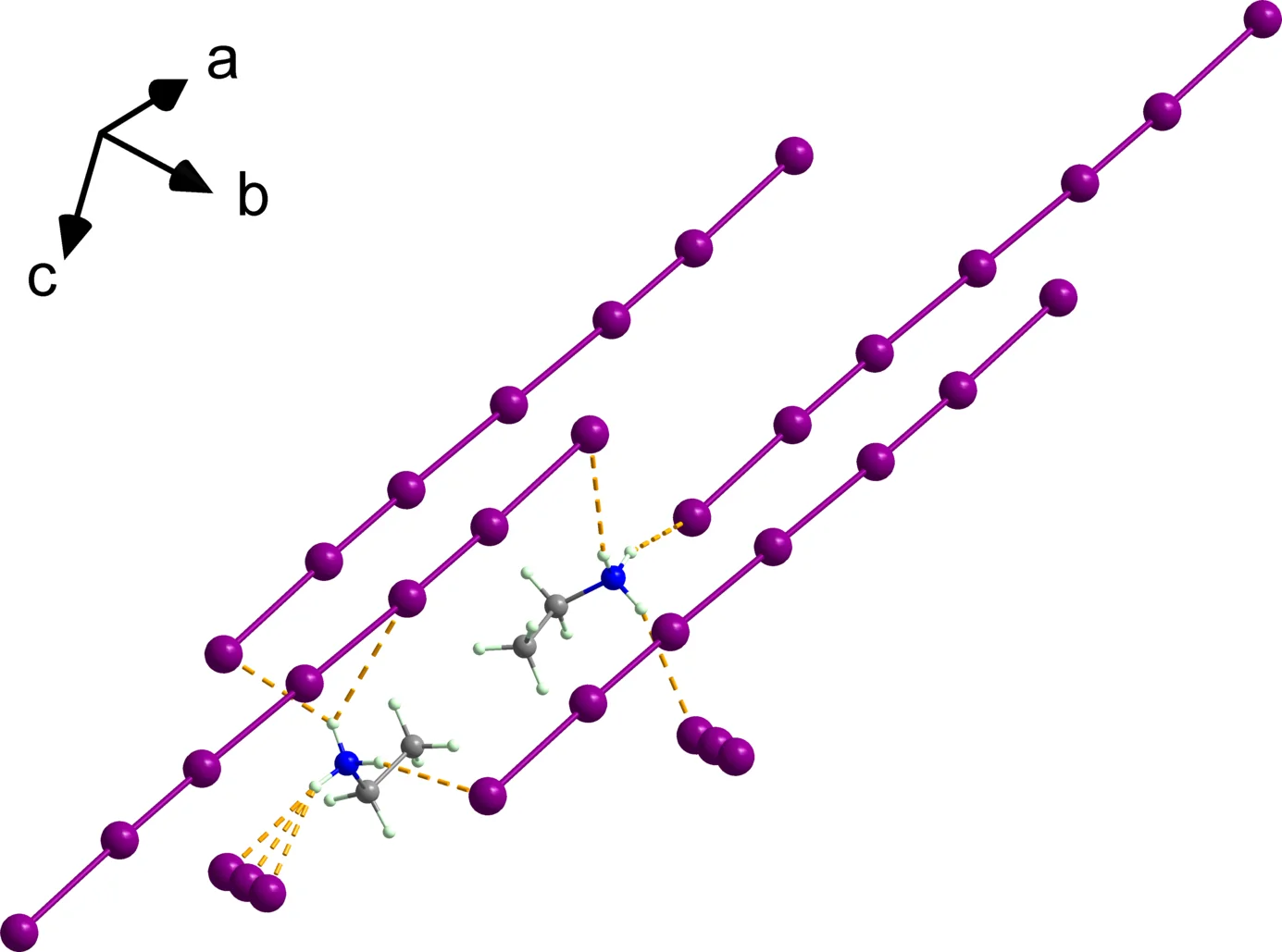
A fragment of crystal structure showing the N—H⋯I hydrogen bonds between ethyl­ammonium cations and both iodide and hepta­iodide anions (N1—C1—C2 cation left; N2–C3—C4 cation right).

**Table 1:** Selected structural parameters (Å, °)of the polyiodide anion

I—I bond	Length	I—I—I	Angle
I1—I2	3.4445 (4)	I1—I2—I3	177.961 (16)
I2—I3	2.7604 (6)	I2—I3—I4	177.540 (16)
I3—I4	3.3747 (5)	I2—I1—I2^i^	180.0

Symmetry code: (i) 2 − *x*, *y*, 1 − *z*.

**Table 2:** Hydrogen-bond geometry (Å, °)

*D*—H⋯*A*	*D*—H	H⋯*A*	*D*⋯*A*	*D*—H⋯*A*
N1—H1*A*⋯I4	0.89	2.77	3.6283 (12)	163
N1—H1*B*⋯I5*A*	0.89	2.62	3.481 (4)	163
N1—H1*B*⋯I5*B*	0.89	2.64	3.521 (4)	170
N1—H1*B*⋯I5*B*^i^	0.89	2.70	3.521 (4)	154
N1—H1*C*⋯I2^ii^	0.89	3.23	3.794 (4)	123
N1—H1*C*⋯I4^iii^	0.89	2.89	3.6283 (12)	141
N2—H2*D*⋯I4^ii^	0.89	2.99	3.811 (8)	155
N2—H2*E*⋯I4^iv^	0.89	2.76	3.597 (9)	158
N2—H2*F*⋯I5*B*^v^	0.89	3.03	3.775 (9)	143

Symmetry codes: (i) 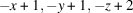; (ii) 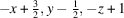; (iii) 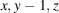; (iv) 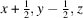; (v) 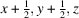.

**Table 3:** Experimental details

Crystal data	
Chemical formula	4C_2_H_8_N^+^·I_7_^3−^·I^−^
*M*_r_	1199.57
Crystal system, space group	Monoclinic, *C*2/*m*
Temperature (K)	293
*a*, *b*, *c* (Å)	23.0274 (12), 7.0212 (3), 9.3382 (5)
β (°)	102.984 (5)
*V* (Å^3^)	1471.19 (13)
*Z*	2
Radiation type	Mo *K*α
μ (mm^−1^)	8.43
Crystal size (mm)	0.32 × 0.11 × 0.06

Data collection	
Diffractometer	XtaLAB Synergy, Dualflex, HyPix
Absorption correction	Analytical [*CrysAlis PRO* (Rigaku OD, 2024 ▸) using a multifaceted crystal model based on expressions derived by Clark & Reid (1995 ▸)]
*T*_min_, *T*_max_	0.043, 0.491
No. of measured, independent and observed [*I* > 2σ(*I*)] reflections	10365, 2106, 1734
*R*_int_	0.061
(sin θ/λ)_max_ (Å^−1^)	0.711

Refinement	
*R*[*F*^2^ > 2σ(*F*^2^)], *wR*(*F*^2^), *S*	0.032, 0.089, 1.04
No. of reflections	2106
No. of parameters	78
H-atom treatment	H-atom parameters constrained
Δρ_max_, Δρ_min_ (e Å^−3^)	0.87, −1.42

Computer programs: *CrysAlis PRO* (Rigaku OD, 2024 ▸), *SHELXT* (Sheldrick, 2015*a* ▸), *SHELXL* (Sheldrick, 2015*b* ▸), *OLEX2* (Dolomanov *et al.*, 2009 ▸) and *publCIF* (Westrip, 2010 ▸).

## supplementary crystallographic information

### Tetrakis(ethylammonium) heptaiodide(3-) iodide Crystal data

**Table:**

4C_2_H_8_N^+^·I_7_^3^^−^·I^−^	*F*(000) = 1064
*M**_r_* = 1199.57	*D*_x_ = 2.708 Mg m^−^^3^
Monoclinic, *C*2/*m*	Mo *K*α radiation, λ = 0.71073 Å
*a* = 23.0274 (12) Å	Cell parameters from 6780 reflections
*b* = 7.0212 (3) Å	θ = 2.6–29.5°
*c* = 9.3382 (5) Å	µ = 8.43 mm^−^^1^
β = 102.984 (5)°	*T* = 293 K
*V* = 1471.19 (13) Å^3^	Plate, clear light orange
*Z* = 2	0.32 × 0.11 × 0.06 mm

### Tetrakis(ethylammonium) heptaiodide(3-) iodide Data collection

**Table:**

XtaLAB Synergy, Dualflex, HyPix diffractometer	2106 independent reflections
Radiation source: micro-focus sealed X-ray tube, PhotonJet (Mo) X-ray Source	1734 reflections with *I* > 2σ(*I*)
Mirror monochromator	*R*_int_ = 0.061
Detector resolution: 10.0000 pixels mm^-1^	θ_max_ = 30.4°, θ_min_ = 2.6°
ω scans	*h* = −32→30
Absorption correction: analytical [CrysAlisPro (Rigaku OD, 2024) using a multifaceted crystal model based on expressions derived by Clark & Reid (1995)]	*k* = −9→9
*T*_min_ = 0.043, *T*_max_ = 0.491	*l* = −12→12
10365 measured reflections	

### Tetrakis(ethylammonium) heptaiodide(3-) iodide Refinement

**Table:**

Refinement on *F*^2^	Hydrogen site location: mixed
Least-squares matrix: full	H-atom parameters constrained
*R*[*F*^2^ > 2σ(*F*^2^)] = 0.032	*w* = 1/[σ^2^(*F*_o_^2^) + (0.0507*P*)^2^] where *P* = (*F*_o_^2^ + 2*F*_c_^2^)/3
*wR*(*F*^2^) = 0.089	(Δ/σ)_max_ = 0.001
*S* = 1.04	Δρ_max_ = 0.87 e Å^−^^3^
2106 reflections	Δρ_min_ = −1.42 e Å^−^^3^
78 parameters	Extinction correction: SHELXL (Sheldrick, 2015b), Fc^*^=kFc[1+0.001xFc^2^λ^3^/sin(2θ)]^-1/4^
0 restraints	Extinction coefficient: 0.0059 (3)
Primary atom site location: dual	

### Tetrakis(ethylammonium) heptaiodide(3-) iodide Special details

**Table:**

Geometry. All esds (except the esd in the dihedral angle between two l.s. planes) are estimated using the full covariance matrix. The cell esds are taken into account individually in the estimation of esds in distances, angles and torsion angles; correlations between esds in cell parameters are only used when they are defined by crystal symmetry. An approximate (isotropic) treatment of cell esds is used for estimating esds involving l.s. planes.

### Tetrakis(ethylammonium) heptaiodide(3-) iodide Fractional atomic coordinates and isotropic or equivalent isotropic displacement parameters (Å^2^)

**Table:**

	*x*	*y*	*z*	*U*_iso_*/*U*_eq_	Occ. (<1)
I1	1.000000	1.000000	0.500000	0.06616 (18)	
I2	0.85877 (2)	1.000000	0.56630 (4)	0.05694 (16)	
I3	0.74738 (2)	1.000000	0.63002 (4)	0.05321 (15)	
I4	0.61406 (2)	1.000000	0.72342 (5)	0.05892 (16)	
I5A	0.500000	0.500000	1.000000	0.094 (2)	0.5
I5B	0.500000	0.4245 (9)	1.000000	0.0607 (8)	0.25
N1	0.61795 (17)	0.500000	0.8233 (5)	0.0533 (10)	
H1A	0.615158	0.612508	0.778332	0.064*	0.5
H1B	0.585030	0.478650	0.855725	0.064*	0.5
H1C	0.622214	0.408841	0.760206	0.064*	0.5
N2	0.9570 (4)	0.5833 (12)	0.6813 (9)	0.100 (3)	0.5
H2D	0.929722	0.579762	0.597037	0.121*	0.5
H2E	0.992363	0.551554	0.665169	0.121*	0.5
H2F	0.958778	0.700361	0.718616	0.121*	0.5
C1	0.6701 (2)	0.500000	0.9487 (6)	0.0639 (14)	
H1D	0.668800	0.388199	1.008890	0.077*	0.5
H1E	0.668800	0.611801	1.008890	0.077*	0.5
C2	0.7280 (3)	0.500000	0.8956 (8)	0.0710 (17)	
H2A	0.725886	0.404839	0.820771	0.107*	0.5
H2B	0.760713	0.472271	0.976485	0.107*	0.5
H2C	0.733746	0.622890	0.855907	0.107*	0.5
C3	0.9410 (5)	0.4524 (12)	0.7824 (13)	0.091 (3)	0.5
H3A	0.969266	0.461066	0.876466	0.109*	0.5
H3B	0.941948	0.323429	0.745838	0.109*	0.5
C4	0.8785 (4)	0.500000	0.8011 (9)	0.100 (3)	
H4A	0.855137	0.548498	0.710193	0.150*	0.5
H4B	0.881024	0.594414	0.876594	0.150*	0.5
H4C	0.860145	0.387021	0.828282	0.150*	0.5

### Tetrakis(ethylammonium) heptaiodide(3-) iodide Atomic displacement parameters (Å^2^)

**Table:**

	*U*^11^	*U*^22^	*U*^33^	*U*^12^	*U*^13^	*U*^23^
I1	0.0375 (3)	0.1007 (4)	0.0587 (3)	0.000	0.0076 (2)	0.000
I2	0.0554 (2)	0.0580 (2)	0.0585 (2)	0.000	0.01484 (16)	0.000
I3	0.0537 (2)	0.0511 (2)	0.0544 (2)	0.000	0.01120 (15)	0.000
I4	0.0477 (2)	0.0455 (2)	0.0861 (3)	0.000	0.02051 (18)	0.000
I5A	0.0704 (13)	0.100 (5)	0.125 (3)	0.000	0.0488 (14)	0.000
I5B	0.0613 (13)	0.064 (3)	0.0609 (14)	0.000	0.0225 (9)	0.000
N1	0.045 (2)	0.056 (2)	0.059 (2)	0.000	0.0106 (18)	0.000
N2	0.075 (5)	0.138 (8)	0.093 (6)	0.018 (4)	0.031 (4)	0.039 (5)
C1	0.054 (3)	0.077 (4)	0.056 (3)	0.000	0.004 (2)	0.000
C2	0.052 (3)	0.069 (4)	0.085 (4)	0.000	0.000 (3)	0.000
C3	0.097 (7)	0.064 (9)	0.106 (7)	−0.006 (5)	0.010 (6)	0.006 (5)
C4	0.130 (8)	0.095 (5)	0.089 (5)	0.000	0.054 (6)	0.000

### Tetrakis(ethylammonium) heptaiodide(3-) iodide Geometric parameters (Å, º)

**Table:**

I1—I2	3.4445 (4)	N2—C3	1.423 (10)
I2—I3	2.7604 (6)	C1—H1D	0.9700
I3—I4	3.3747 (5)	C1—H1E	0.9700
I5A—I5B	0.530 (6)	C1—C2	1.522 (9)
I5A—I5B^i^	0.530 (6)	C2—H2A	0.9600
I5B—I5B^i^	1.061 (13)	C2—H2B	0.9600
N1—H1A	0.8900	C2—H2C	0.9600
N1—H1B	0.8900	C3—H3A	0.9700
N1—H1C	0.8900	C3—H3B	0.9700
N1—C1	1.478 (6)	C3—C4	1.525 (11)
N2—H2D	0.8900	C4—H4A	0.9600
N2—H2E	0.8900	C4—H4B	0.9600
N2—H2F	0.8900	C4—H4C	0.9600
			
I3—I2—I1	177.961 (16)	C1—C2—H2A	109.5
I2—I3—I4	177.540 (16)	C1—C2—H2B	109.5
I5B—I5A—I5B^i^	180.0	C1—C2—H2C	109.5
I5A—I5B—I5B^i^	0.000 (2)	H2A—C2—H2B	109.5
H1A—N1—H1B	109.5	H2A—C2—H2C	109.5
H1A—N1—H1C	109.5	H2B—C2—H2C	109.5
H1B—N1—H1C	109.5	N2—C3—H3A	109.9
C1—N1—H1A	109.5	N2—C3—H3B	109.9
C1—N1—H1B	109.5	N2—C3—C4	109.0 (7)
C1—N1—H1C	109.5	H3A—C3—H3B	108.3
H2D—N2—H2E	109.5	C4—C3—H3A	109.9
H2D—N2—H2F	109.5	C4—C3—H3B	109.9
H2E—N2—H2F	109.5	C3^ii^—C4—C3	25.3 (6)
C3—N2—H2D	109.5	C3—C4—H4A	109.5
C3—N2—H2E	109.5	C3^ii^—C4—H4A	100.3
C3—N2—H2F	109.5	C3^ii^—C4—H4B	91.8
N1—C1—H1D	109.5	C3—C4—H4B	109.5
N1—C1—H1E	109.5	C3—C4—H4C	109.5
N1—C1—C2	110.9 (5)	C3^ii^—C4—H4C	134.0
H1D—C1—H1E	108.0	H4A—C4—H4B	109.5
C2—C1—H1D	109.5	H4A—C4—H4C	109.5
C2—C1—H1E	109.5	H4B—C4—H4C	109.5
			
N2—C3—C4—C3^ii^	−39.0 (8)		

Symmetry codes: (i) −*x*+1, −*y*+1, −*z*+2; (ii) *x*, −*y*+1, *z*.

### Tetrakis(ethylammonium) heptaiodide(3-) iodide Hydrogen-bond geometry (Å, º)

**Table:**

*D*—H···*A*	*D*—H	H···*A*	*D*···*A*	*D*—H···*A*
N1—H1*A*···I4	0.89	2.77	3.6283 (12)	163
N1—H1*B*···I5*A*	0.89	2.62	3.481 (4)	163
N1—H1*B*···I5*B*	0.89	2.64	3.521 (4)	170
N1—H1*B*···I5*B*^i^	0.89	2.70	3.521 (4)	154
N1—H1*C*···I2^iii^	0.89	3.23	3.794 (4)	123
N1—H1*C*···I4^iv^	0.89	2.89	3.6283 (12)	141
N2—H2*D*···I4^iii^	0.89	2.99	3.811 (8)	155
N2—H2*E*···I4^v^	0.89	2.76	3.597 (9)	158
N2—H2*F*···I5*B*^vi^	0.89	3.03	3.775 (9)	143

Symmetry codes: (i) −*x*+1, −*y*+1, −*z*+2; (iii) −*x*+3/2, *y*−1/2, −*z*+1; (iv) *x*, *y*−1, *z*; (v) *x*+1/2, *y*−1/2, *z*; (vi) *x*+1/2, *y*+1/2, *z*.
